# Coding and educational robotics with peers: The C0D1NC experience to foster inclusion

**DOI:** 10.3389/frobt.2022.825536

**Published:** 2022-09-16

**Authors:** Michela Ponticorvo, Franco Rubinacci, Elena Dell’Aquila, Davide Marocco

**Affiliations:** Natural and Artificial Cognition Lab “Orazio Miglino”, Department of Humanities, University of Naples “Federico II”, Naples, Italy

**Keywords:** educational robotics, sociometric tools, social networks, assessment, students’ groups, coding, inclusion

## Abstract

In the present paper, the experience of the C0D1NC project (Coding for inclusion) is described. In this project an innovative methodology based on peer-education is the core of the educational approach. High school students become “teachers” as they are trained to teach coding and robotics to younger students. This approach favors inclusion and digital inclusion. To affirm this, we evaluated different aspects: relations between peers, perceived self-efficacy, and attitude towards technology at the beginning of activities (pre-test) and the end (post-test). Results indicate that this approach can be effective to favor personal growth, improved relations between peers, and increased self-efficacy too.

## 1 Introduction

In recent years, the theme of digital inclusion is gaining a wider space, as digital tools are entering every context of human life at every age and this diffusion has become even wider during the COVID-19 pandemic (Vargo et al., 2021). It is therefore crucial that a wide range of people can master digital tools. Digital technologies allow us to work from home, acquire new skills flexibly, and get informed on what is going on worldwide instantly. In brief, the digital transition has offered many new opportunities not equally exploitable for everyone. Some people cannot access the digital world, others may not be able to use technologies thoroughly or conscientiously, others can be impeded in participating in this digital revolution by sensory impairment, learning disabilities and so on (Warschauer & Matuchniak, 2010; Alper & Goggin, 2017). Indeed, digital inclusion is nowadays crucial, and it is supported by European digital strategy that defines it as the “EU-wide effort to ensure that everybody can contribute to and benefit from the digital world” (EU Digital Inclusion Strategy, 2021). Therefore, the intervention to improve digital inclusion is focused on the accessibility of ICT, the development of assistive technologies, the empowerment of skills and digital skills, and the increase of social inclusion (ibidem). These latter two directives become particularly relevant when we talk about already marginalized conditions and contexts where social exclusion starts from school, leading to school drop-out and later impacts on careers (De Witte et al., 2013; Balenzano et al., 2019; Scandurra et al., 2021). In these contexts, digital inclusion can become a vehicle for social inclusion (Atkinson et al., 2002; Warschauer, 2004; Rawal, 2008; Nguyen, 2020), as nowadays an important part of social inclusion in societies is e-inclusion (Pentzaropoulos and Tsiougou 2014; Vassilakopoulou & Hustad, 2021). In virtue of this connection, interventions should have the twofold goal to favour the acquisition of technical competences related to ICT and, on the other side, to foster social inclusion, increasing the participation of disadvantaged and marginalized people (Allman, 2013). Moreover, in order to have stronger effects, it is effective to start these interventions since school period to promote, at the same time, the acquisition of the basics of digital tools and increase the motivation to stay at school and go on in the educational pathway (Kim et al., 2021).

In the present paper we describe and discuss an experience we have run in the EU-funded project C0D1NC that explored how coding and robotics teaching and learning could affect social relations and personal aspects favoring inclusion as self-efficacy (Bandura, 1989; Burke, 2020). The educational core approach for C0D1NC was peer-education as high school students were trained to teach coding and robotics to younger students. We investigated if this double-role can have some beneficial effects in acquiring technical skills and to foster inclusion. From our previous studies, we had indications that robotics and tangible materials can be effective in promoting positive relations between peers (Ponticorvo et al., 2020a; Ponticorvo et al., 2020b).

We tried to answer these research questions: is peer-education approach applied to coding and robotics effective in promoting social relations? Do coding and robotics activities impact on group variables, such as social links and on individual variables such as self-efficacy? The paper is organized as follows: we introduce the educational approaches that informed the C0D1NC Project, we describe in detail the method we used and then we report results.

## 2 C0D1NC project: Educational approaches

In the C0D1NC Project we select educational approaches and teaching strategies that enable the active involvement of learners in productive educational pathways by creating new knowledge and meaningful exchange sharing their own experiences. These approaches and strategies include collaborative learning (Resta, & Laferrière, 2007), peer education (Green, 2001), effective applications of creativity to education (Ponticorvo et al., 2020b), introduction to real-life situations and hands-on experiences for reflection and building new ways of thinking. Collaborative learning theory entails peer-to-peer learning that nurtures deeper thinking among learners. Moreover, according to the constructionist approach (Harel & Papert, 1991), learners learn best by making tangible objects within authentic learning opportunities that allow for a guided, collaborative process that integrates peer feedback.

All these strategies are predominantly ways of supporting the learner in actively making improvements for herself. There is a broad recognition that the critical elements of effective learning strategies are the will to engage in learning (Blumenfeld et al., 2006) and within activities that pose a challenge and the skills associated with coming to a deeper understanding of content.

Experiences of peer-to-peer education are helpful to fill the students’ gaps in storing and delivering the insights of a particular subject matter to gather the aspects that they did not grasp previously (Heller et al., 2000; da Silva et al., 2021). They can join the discussion more responsibly with their peers to work out jointly more information related to specific topics and propose new ways to address it to the group members.

The constructivist approach (Papert & Harel, 1991) emphasizes learners’ agency as active participants in constructing their own learning rather than just taking in information passively. This approach argues that learning occurs more effectively whether learners are involved in producing tangible and shareable objects. By experiencing the world and reflecting upon those experiences, learners build their own mental models to understand the world around them and integrate new information into their pre-existing knowledge. Papert was the first to introduce technology and educational robotics into classrooms as a learning tool for illustrating concepts (math, science, and technology). The iterative process involved in building a robot allows students to experience the effect of their experimentation tangibly, learn from mistakes, identify those mistakes, try until they have a functioning robot, and develop critical thinking within collaborative environments. Indeed, this approach crucial for enhancing positive technological fluency has led to the development of the STEAM (science, technology, engineering, arts, and mathematics) centred approaches to student learning and engagement (Hamir et al., 2015).

Moreover, some experiences have proven to effectively promote different skills, both hard and skills that are useful for inclusion and digital inclusion. For example, Educational Robotics (ER) can be an effective teaching and learning tool (Miglino et al., 1999) as it is focused on technical knowledge such as mathematics, computer science, physics (Lindh and Holgersson, 2007; Williams et al., 2007; Nugent et al., 2009). It is also fit in soft skills training, including thinking skills and problem solving approaches (Hussain et al., 2006; Sullivan, 2008; Mikropoulos and Bellou 2013; Atmatzidou and Demetriadis, 2016; Gabriele et al., 2017).

Educational Robotics (ER) implies an integrated approach in which different competencies and skills are needed to promote interest and curiosity in scientific issues, mainly digital technologies (Aris and Orcos, 2019). Kandlhofer and Steinbauer (2016) indicate that ER leads to a high achievement in social skills and self-esteem in students that turns into greater motivation (Bazylev et al., 2014), which is a pivotal element in enhancing learning. At school, a low level of social inclusion can dramatically affect relevant phenomena, including school drop-out (Frostad et al., 2015; Ricard and Pelletier, 2016). The relations with peer affect social inclusion at school that, in turn, can be a protective factor in respect to school drop-out (Balenzano et al., 2019). It is interesting to underline that ER activities need to be run in groups, thus promoting collaborative work and collaborative learning (Denis and Hubert, 2001). Some previous work run by the authors (Ponticorvo et al., 2020a) have indicated that ER can be effectively used to promote knowledge related to STEM and to enhance different skills such as computational thinking, problem-solving, complex systems management and collaborative learning, and positive and collaborative relations between students (Rubinacci et al., 2017a; Rubinacci et al., 2017b; Truglio et al., 2018a; Truglio et al., 2018b). ER allows to establish a relation of interdependence among students, who must achieve a common goal (Burbaite et al., 2013; Kamga et al., 2016), coordinate their efforts, learn to divide their tasks, and learn to complete the task, considering other group members. This represents a chance also for students with a low level of inclusion to participate in a group activity, thus improving their relationships with other students.

The peer education approach can be effectively used to increase students’ participation and retention. It rests on the premise that equal participatory dialogue can drive the desired behavioral changes (McKeganey, 2000). Young people are considered the experts in their own lives; thus, they are the best starting point in any learning process. Indeed, this approach aims to train well-motivated young people in undertaking organized educational activities with their peers (similar in age, interest, background, etc.) over some time, intended to develop their knowledge, skills, and attitudes. The benefits of peer learning for students entail the opportunity to improve attitudes toward more personalized and engaging learning and foster collaborative and cooperative learning, leading to more significant accomplishments.

These approaches were the core of C0D1NC Project. In the next section, we will describe this project in detail.

## 3 C0D1NC project: Educational activities

The C0D1NC “Coding for Inclusion” Project is a European project funded under ERASMUS+ KA3 “Social Inclusion through Education, Training and Youth”. This programme aimed at fostering STEM education of disadvantaged youth through an inclusive educational approach based on a peer-learning pedagogical method for formal and non-formal educational contexts in Europe. The “Coding for Inclusion” was run in 5 European countries, namely Belgium, Cyprus, Germany, Spain, and Italy; of this latter we will describe the trial and results in detail. The project objectives were twofold and addressed both technical skills and soft skills (Dell'Aquila et al., 2017), remarkably increasing STEM education in disadvantaged areas and favouring collaborative competences, problem-solving, and creativity. For the Italian side, the project took place in Naples and its surroundings, an area in Southern Italy that is highly affected by school drop-out resulting in threats at the social level (O'Higgins et al., 2007; Odoardi, et al., 2021). As described in the introduction, ER can effectively achieve this twofold goal; the C0D1NC project goes a step forward marrying ER with a peer-education approach and investigating if this plus could be impact on social relations. After a training phase that involved the staff and covered both the toolkit and the methodology, the trial phase in Italy started. In the next sub-section we introduce the details on C0D1NC Project: educational activities.

### 3.1 Materials and method

#### 3.1.1 Participants

The trial phase involved teachers and pupils from the second year of high school (corresponding to the 10th grade) with socio-economic specialization and teachers and pupils from 4 secondary school (2 at the first year and 2 at the second year). A total of 90 pupils and seven teachers were involved. Both schools were identified in disadvantaged areas with low socio-economic level and at considerable risk of social exclusion and drop-out in Naples. The schools were selected by researchers, whereas the school selected the classes and the students to be involved in the activities. The trials lasted 5 months between January and May 2019.

In January 2019, the trial began with the kick-off meeting in which teachers and students were informed about C0D1NC aims, objectives and methodology. Parents of participants gave the informed consent at the beginning of the school year. Questionnaires to evaluate the activities were administered to the group involved in the trials at the start (pre-test) and at the end of the activities (post-test). Alongside the group involved in the trials, we considered a control group who did not take part in C0D1NC activities. On these groups we run different assessments for different groups of participants: for social relations we used the sociometric test, as already done in our previous study (Ponticorvo et al., 2020a); for individual variables we used scale for self-efficacy, attitudes toward students and towards technology that are described in next sub-sections.

#### 3.1.2 Sociometric test

Students attending high schools were administered the sociometric test developed by Jacob L. Moreno (Moreno, 1941, 1951), widely employed to analyze interpersonal relationships within a group, thus identifying the social position of each group member. The sociometric test proposed to the students explored 1) affective-relational aspects and 2) functional aspects: the first one refers to the affective relationships established between the members of a group and the psychological affinities of the group members; the second one is related to the organization of the group and is aimed at understanding the relationships to achieve a common goal.

Indeed, the sociometric test can be used to explore and delineate different kinds of relations if different questions are used. If the goal is to explore affective-relational aspects, questions will be like “Who would you go to the cinema with?” whereas, for functional aspects, an example would be: “Who would you choose as a companion for homework?”. For this study in particular researchers asked, for the affective-relational aspect: “Who would you like as room-companion for a school trip?”; for functional aspects they asked: “Who would you choose as a companion for a school workgroup?”

The data from the sociometric test can be reported in a graph called sociogram, which represents the relationship between people with nodes and lines. Nodes represent group members, whereas lines indicate if a relation is present and of what kind this relation is. The arrows indicate the direction of the relationship. Lines with one arrow represent unidirectional relationships, whereas those with two arrows indicate a bidirectional relationship. Sociometric data can also be arranged in a double-entry table named sociomatrix. From the sociogram and the sociomatrix, rich information can be gained on the group structure and members’ links, including the number of choices and rejections that each member of the group has received; the degree of reciprocity of choices and rejections; the difference between ignored, rejected, isolated and popular subjects.

From the sociometric test, indexes can be derived, and statistical techniques can be applied to them to get a more significant amount of data on group interaction and dynamics (Ponticorvo et al., 2020a).

The Sociometric Affective Test was administered to all members of the class group (primary and secondary), with and without disabilities, at the beginning (pre) and the end (post) of the experimentation. The Sociometric Group Organization Test was administered to all members of the class group (primary and secondary), with and without disabilities, at the beginning (pre) and at the end (post) of the experimentation.

#### 3.1.3 Scales on self-efficacy, attitudes toward students and towards technology

Together with the sociometric test, which was used to explore social relations, we also investigated personal variables. In more detail, we administered the perceived Self-Efficacy scale (Caprara, 2001) and the Teachers’ Attitudes Toward Students Scale -TATS (Ng, 2002) to students. The first one is a widely used scale to assess self-efficacy (ref), a psychology construct that is related to “know to be able to do” related with academic motivation, achievement and many other variables that are relevant to facilitate inclusion (Block et al., 2010; Kiel et al., 2020; Hosford and O'Sullivan, 2016; Lent et al., 1984; Bahcekapili and Karaman, 2020). Self-Efficacy Students Scale was administered to all members of the class group (primary and secondary), with and without disabilities, at the beginning (pre) and the end (post) of the experimentation.

The second scale investigates teachers’ attitudes towards students from two different approaches: conservative and autocratic versus liberal and democratic. TATS is a self-reporting measure, and participants must express and evaluate their degree of agreement or disagreement with each item of the test, using a 5-point Likert scale with “1”—“Strong disagreement” and “5”—“Strongly agree.” The scale consists of 14 items divided into two groups, each of seven items. One set of elements measures the conservative-autocratic attitude, whereas the other measures the liberal-democratic attitude. This scale was administered to students only at high school, to investigate what a teacher should be like from their point of view. TATS Scale was administered to the students at the secondary school, with and without disabilities, at the beginning (pre) and at the end (post) of the experimentation; it was not proposed to younger students.

We administered to teachers 2 assessment scales to investigate the level of self-efficacy and the ability to integrate technology into teaching: the Teacher Self-Efficacy Scale- SAED (Tschannen-Moran et al., 1988; Tschannen-Moran & Hoy., 2001; for the Italian validation Biasi et al., 2014) and the Intrapersonal Technology Integration Scale–ITIS (Niederhauser & Perkmen, 2008; for the Italian validation Benigno et al., 2014). The scale consists of 21 Items and takes 10–15 min to complete, it was administered to all teachers involved in the activities (first and second degree) at the beginning and end (before and after) of the trial.

### 3.2 Tools for coding and educational robotics

The following tools for coding and educational robotics were used for the study reported here: *Scratch* and *Makey Makey*.

*Scratch* (Resnick et al., 2009; https://scratch.mit.edu/) is a free programming language that allows the program of interactive stories, games, and animations. Scratch also allows sharing creations among community members. Scratch is an excellent tool to promote creative thinking, computational thinking, systems reasoning and collaborative working. It was specifically designed and developed for young students, for the 8–16 age group.

*Makey Makey* (https://makeymakey.com/) is a kit that allows to make many things interactive, thanks to the connection, using alligator clips contained in the kit. This way, the Makey Makey kit allows to transform anything into a touch controller. For example, it allows to make fruit play, associate voice signals with cards, objects, make a controller with what you have on hand. It can be done easily: touching a Makey Makey object allows you to generate an electrical signal transmitted via the cable. The board has inputs that send the signal to the USB port for connection with the PC or Tablet. The inputs allow you to control some keyboard functions: the four direction arrows, the space bar, the letters W, A, S, D, F and G and mouse clicks. It can be used to build many robotics applications in education contexts (Marín-Marín et al., 2020).

### 3.3 Procedure

After the kick-off meeting where assessment tools were administered, C0D1NC activities began according to a defined schedule. Students and teachers at the high school were trained to use the technological tools and teach them to their younger peers. At the beginning of activity at the high school, there were 24 students (22 females and 2 males) at the conclusion of the activities 16 students (14 females and 2 males).

## 4 Results

### 4.1 Sociometric analysis

Starting from the sociomatrices, we built the sociograms and calculated various indexes described by Garcia-Magarino et al. (2019), with the software Gephi, an open-source software for analysing and visualising social networks (Bastian et al., 2009). The groups receiving the intervention were compared with a control group that was not involved in C0D1NC activities. We report here some relevant examples, as we found similar results for all groups. The complete series of sociograms and indexes is reported in Supplementary Appendix S1.

Starting from metrics about selections and rejections, we calculated:

1) the *coherence* index, consisting of the ratio between reciprocal selections and the selections received by other students.

$$c=\;\frac{s_{bid}}{s_{rec}}$$

2) the *density* index measures the percentage of relations (either reciprocal or not) among the number of possible paired combinations. In other words, it is the proportion of realized edges out of all possible edges: $e_{a}$ is the number of actual edges whereas n (n−1) is the maximum number of directed edges calculated on the number of nodes n.

$$d=\frac{e_{a}}{n\;\left(n-1\right)}$$

Moreover, we represented the groups using sociograms, reported in the next figures.

Here we report the sociometric test related to the class involved in the project: a class group of 16 students, 14 females and 2 males with an average age of 15.25 years of a second class of the Liceo “Margherita di Savoia” in Naples. In the class group, there were two subjects with Special Educational Needs. In this school, the control group was formed by 11 participants, 9 females and 2 males. The control group was involved in group activities proposed by teachers. Sociograms and indexes are reported both for affective/relational dimension and group organization dimension.

In the tables, positive connections refer to the selections, the choices, in the sociometric test and negative connections refers to the refusals, to the negative replies in the sociometric test. Average refers to the average edges for the nodes in the group; density and coherence are defined above.

Nodes refers to the numbers of individuals who were in the classroom and participated to the sociometric test (the number does not change between pre-test and post-test), the letter refers to a code to identify the same node (participant) between pre-test and post-test. The node has a colour and a dimension that is proportional to the number of received selections: more selections correspond to a wider diameter and a darker colour. Green is used for affective/relational dimension and blue for organizational dimension. Arrows can indicate reciprocal selection (in this case the ends are equal) or mono-directional selection. These details are defined in the Gephi software interface.

**FIGURE 1:**
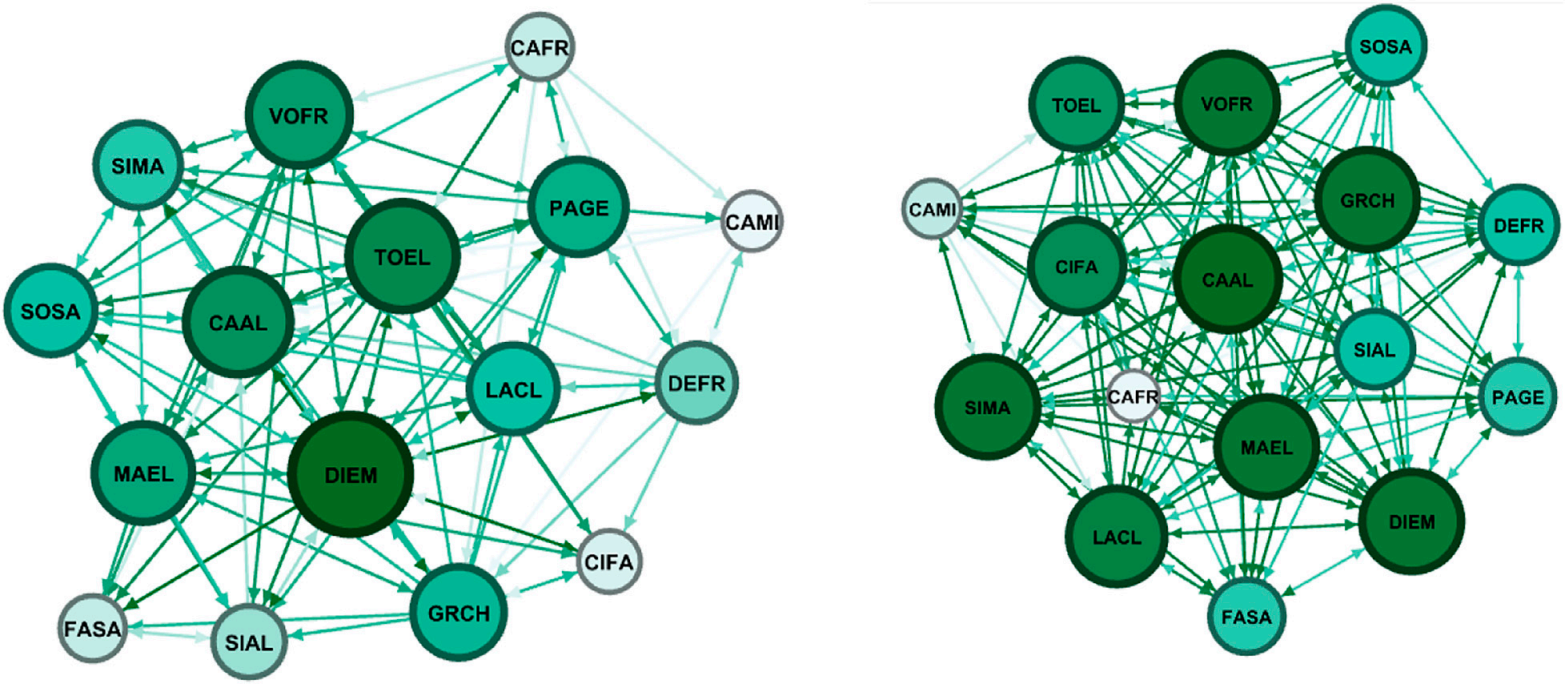
Pre-Test (on the left) and post-test (on the right) Sociograms on affective/relational dimension for the group involved in the trial.

In Figure 1 and Table 1, we report the sociograms built on affective/relational dimension for the group involved in the trial before the intervention and after. Sociograms showed that the group becomes much more connected with a relevant increase of positive connections and a decrease of negative ones. The number of edges increases of 54% and positive connections, that indicate selections are more than doubled. At the same time, negative connections, indicating refusals, decrease.

**TABLE 1:** Sociometric index calculated for the group involved in the trial (affective/relational).

	Nodes	Edges	Average	Density	Coherence	Positive connections	Negative connections
Pre test	16	123	7,7	0,51	0,68	73	50
Post test	16	189	11,8	0,79	0,74	162	27
		+54%				+122%	−46%

**FIGURE 2:**
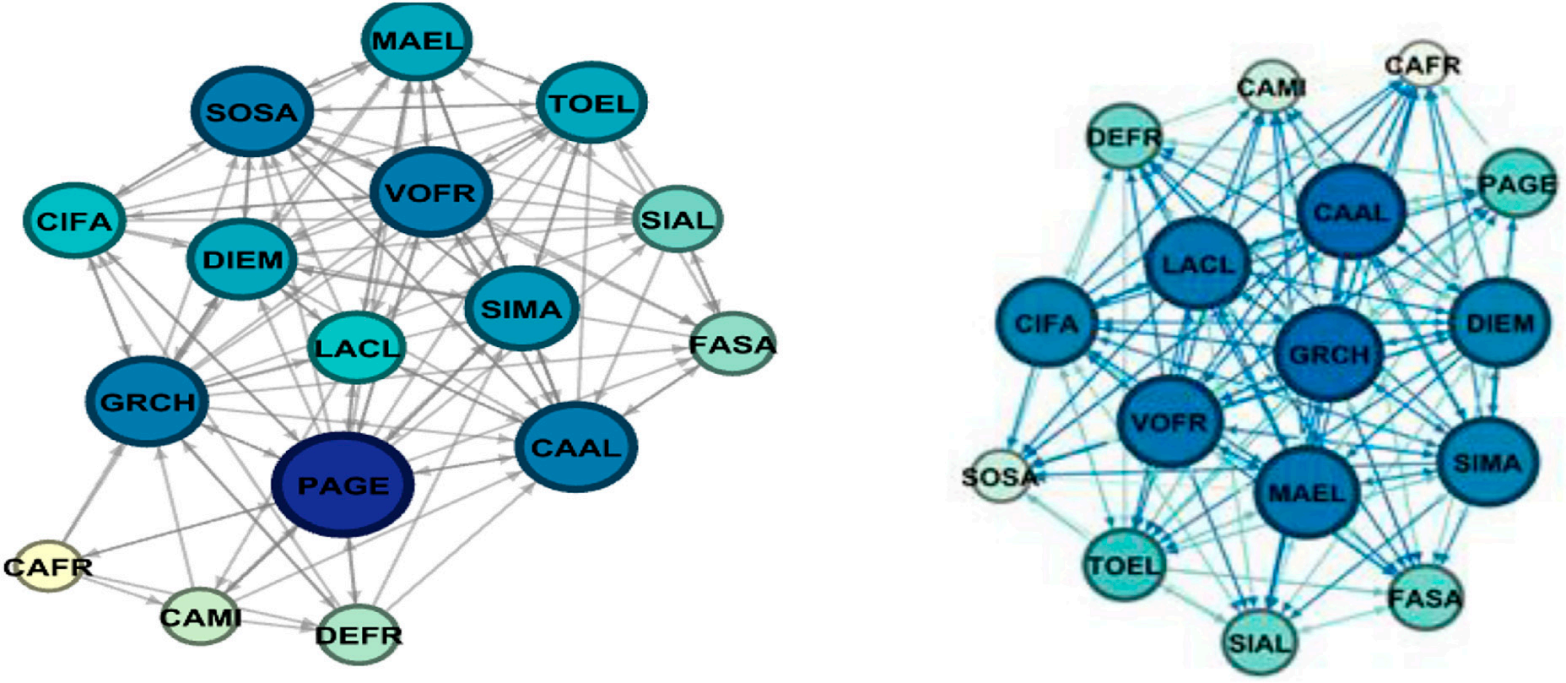
Pre-Test (on the left) and post-test (on the right) Sociograms on group organization dimension for the group involved in the trial.

In Figure 2 and Table 2, we report the sociograms built on group organization dimension for the group involved in the trial before the intervention and after. For this group, also on the organization dimension, we observe a relevant increase of positive connections and a decrease of negative ones, even more evident than on the affective/relational dimension.

**TABLE 2:** Sociometric index calculated for the group involved in the trial (group organization).

	Nodes	Edges	Average	Density	Coherence	Positive connections	Negative connections
Pre test	16	118	7,4	0,49	0,56	74	44
Post test	16	178	11,1	0,74	0,69	165	13
		+51%				+123%	−70%

This group, involved in the C0D1NC Project activities, show much more selections, positive connections that reflect a change in the relation dynamic in a positive direction.

**FIGURE 3:**
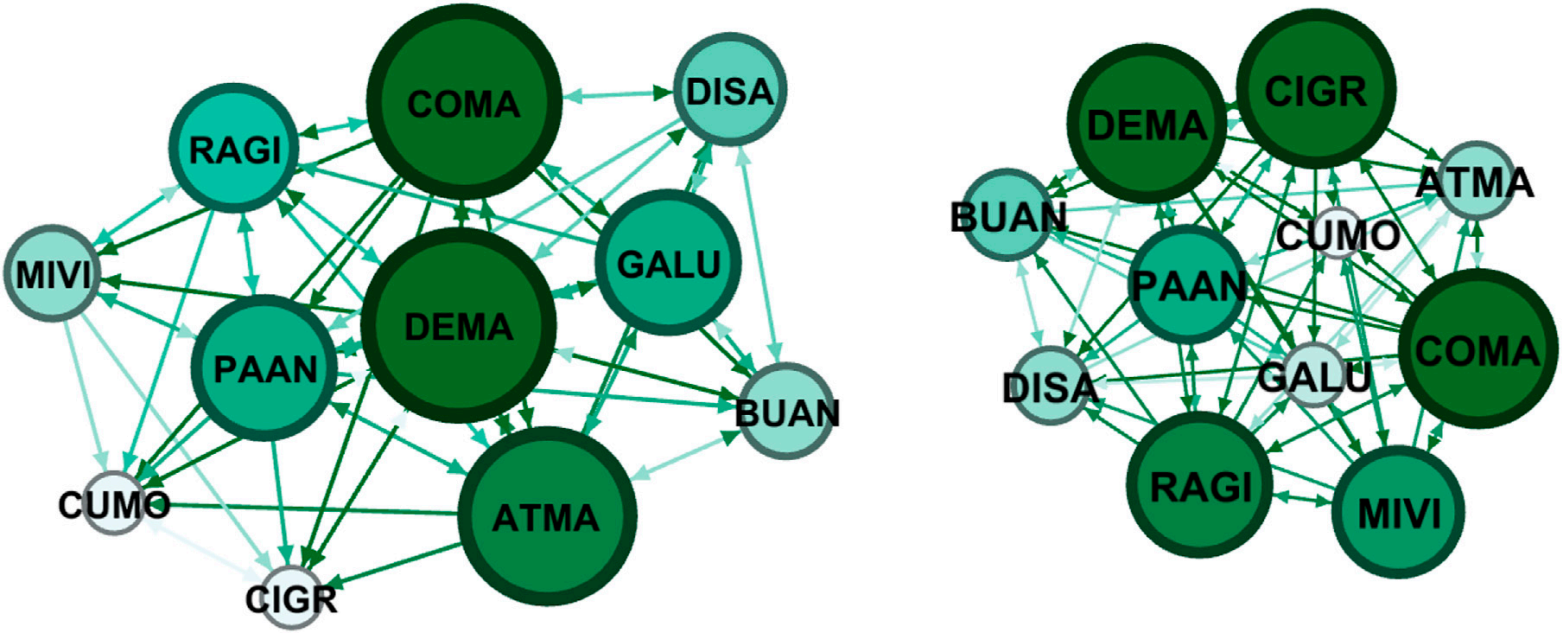
Pre-Test (on the left) and post-test (on the right) Sociograms on affective/relational dimension for the control group.

In Figure 3 and Table 3 there are the sociograms built on affective/relational dimension for the control group before the intervention and after. For this group, there is a slight increase of positive connections whereas negative connections remain the same.

**TABLE 3:** Sociometric index calculated for the control group (affective/relational).

	Nodes	Edges	Average	Density	Coherence	Positive connections	Negative connections
Pre test	11	66	6	0,6	0,73	59	7
Post test	11	72	6,5	0,65	0,64	65	7
		+9%				+10%	=

In this group that is not involved in C0D1NC Project activities, it is possible to observe a sociometric picture that remains essentially the same between pre and post-test.

**FIGURE 4:**
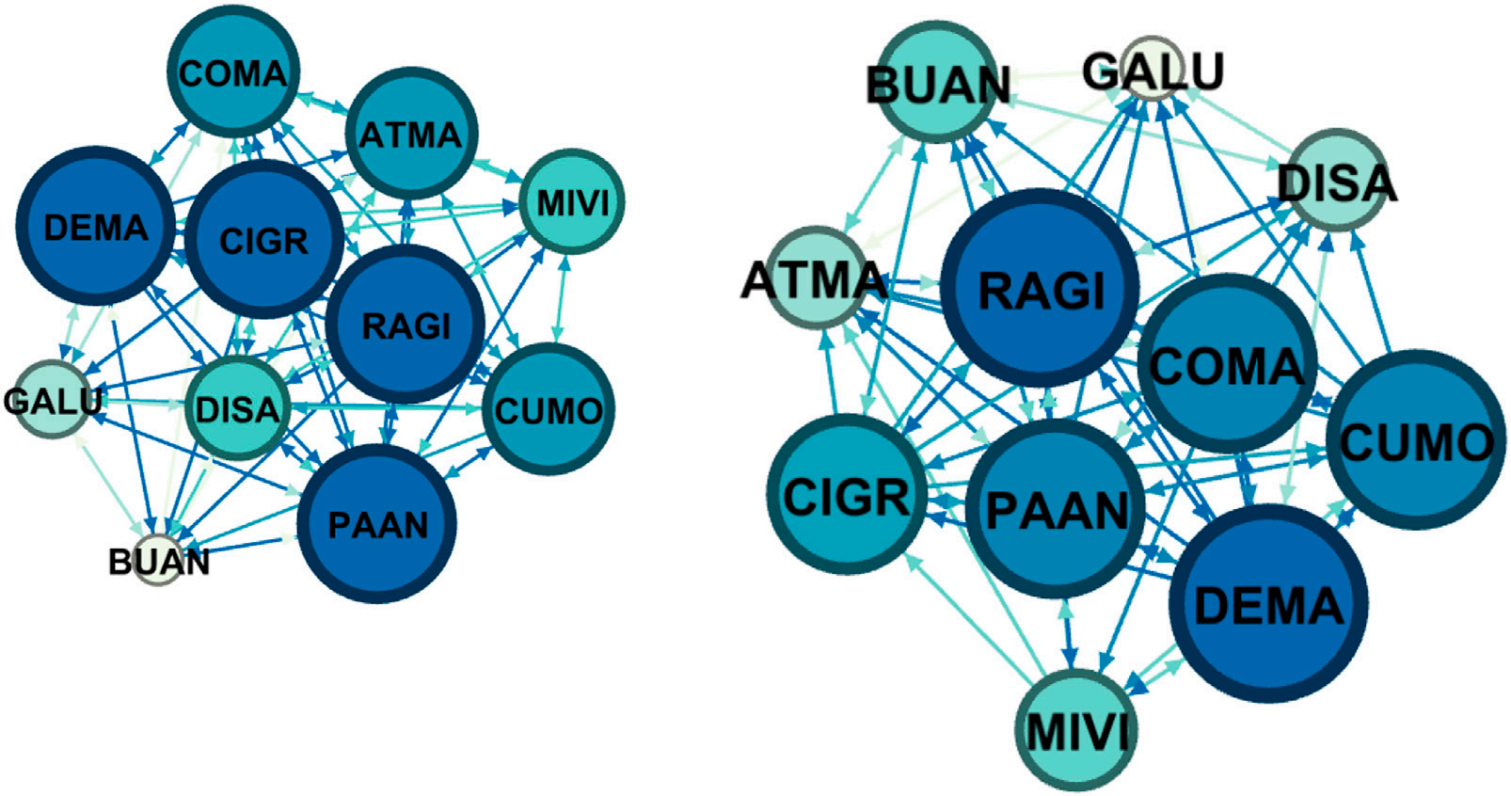
Pre-Test (on the left) and post-test (on the right) Sociograms on group organization dimension for the control group.

Figure 4 and Table 4 represent sociograms and indexes for group organization dimension in the control group before the intervention and after. We observe that there is a decrease of both positive and negative connections, even if slight.

**TABLE 4:** Sociometric index calculated for the control group (group organization).

	Nodes	Edges	Average	Density	Coherence	Positive connections	Negative connections
Pre test	11	96	8,77	0,87	0,9	72	25
Post test	11	80	7,3	0,7	0,75	63	17
		+17%				−12%	−18%

For the group organization dimension, the sociometric test leads to similar results both at pre-test and post-test, indicating a comparable group situation.

Summarizing, sociograms and indexes show a significant increase in positive relationships between students between the pre and post-test for participants involved in C0D1NC Project activities, as pictured in Figure 1. Connections in general increased by 54% between pre and post-test (see Table 1), positive ones (choices) were more than doubled, whereas negative ones (refusals) halved.

In Figure 2 we can see the graphic representation (sociogram) of the results between the pre and post-test of the sociometric test regarding the aspect of the group organization. Also, in this case we can observe that connections and particularly positive connections increased (see Table 2).

In the control group, on the other side, there is a slight increase of connection between pre and post-test, and the increase covered negative connections, whereas there was a slight increase in positive ones (Figures 3, 4 and Tables 3 and 4).

Participating in C0D1NC Project activities led to more selections and more positive connections, indicating a measurable change in group situation, whereas the control group remained substantially the same in sociometric terms.

We then compared the results obtained with the scales on self-efficacy and TATS. We compared the groups involved in the interventions and control groups about the measures on self-efficacy and attitude toward students. We used the non-parametric Wilcoxon test for paired samples. We observed no statistically detectable difference except for self-efficacy in the group of high school that were the ones who also covered the teacher role (Z = - 2.94 *p* < 0.05).

These results indicate that the approach to Educational Robotics, together with favouring the acquisition of basic skills in coding and robotics as showed in previous studies, helped to stimulate positive group dynamics that can be protective against school drop-out. In contrast, it did not have effects on attitude towards teachers.

It is interesting to note that there was an increase in self-efficacy for the group that participated in the teaching of younger students according to a peer-education approach: covering the new role of teacher led to increase individual dimensions as well as group ones.

## 5 Discussion and conclusion

The C0D1NC project has been an opportunity to experiment an innovative methodology to acquire and strengthen coding and computational thinking competences together with a chance for personal growth. Students and teachers have shown constant interest in the proposed activities.

Of particular importance was the role that high school students had to assume after the project’s first phase, namely the possibility of becoming “teachers” of the course for younger students.

The choice to conduct the project in a context where we find socio-economic difficulties and a high risk of social exclusion was challenging. Working with young people who often must face family, school, social problems allowed us to explore the potential of Educational Robotics in favouring inclusion and digital inclusion.

C0D1NC was an excellent training and personal growth opportunity for high school students, not only for having enriched their cultural background but even more for having strengthened the level of self-efficacy and cohesion within a group of work.

The reported results support the use of Educational Robotics with a peer-education approach to improve social relations among students who have participated in C0D1NC activities. Moreover, according to the constructivist approach, these activities allow to build new relations with their peers. Adopting a peer-education approach allowed to involve students, in especially at high school, in a way that made them become the leading characters for digital and social inclusion addressed to younger students. Even if the results we have described cannot be extended to the general population at statistical level, as we have worked on small and selected samples, the comparison between the conditions before and after the intervention support our hypothesis that peer-education approach applied to coding and robotics is effective in promoting social relations. Moreover, focusing on coding and robotics activities has proved to impact on group variables, and individual variables such as self-efficacy, which, in turn, can promote social inclusion.

C0D1NC activities led to act in an interdependent way, whereas in the curricular activities this doesn’t always happen. This can be explained considering the change of the perspectives and how students relate to each other in this different context.

Together with the effect on the relationship, the described results support the idea that acting as a teacher for the young participants can also increase the level of self-efficacy, thus having a positive effect on individual variables.

The traditional focus of Educational Robotics activities, which revolve around technical skills acquisition, is becoming wider as ER can be also a vehicle for interventions on psychological and relational aspects. ER can help to develop soft skills, like teamwork, and improve their relationships: this issue is receiving more and more attention (Screpanti et al., 2021) and seems a very promising practice to be applied in schools.

On the first side, as highlighted by the recent review by Tzagkaraki et al. (2021), educational robotics can help transforming traditional teaching and learning practices to better respond to modern educational challenges.

ER can offer useful tools even if its use opens new challenges and it does not always lead to positive results for improving learning (Xia and Zhong, 2018): some researchers have explained this evidence by referring to teachers’ attitudes towards ER in particular and technology in general, or to reduced expertise in coding, informatics and related subjects (Lathifah et al., 2019).

The approach we have proposed in the C0D1NC project and in the present paper, opens new chances as students themselves are invited to become teachers for younger students. Peers are able to promote a collaborative environment where robotics activities are followed with motivation and where a broad participation can happen (Anwar et al., 2019).

This collaborative environment gets strength from the relations’ network, as hinted at before, which becomes crucial for inclusion and related psycho-social construct such as empathy, prosocial behavior, and positive development especially in school context (Spinrad and Eisenberg, 2014).

Using Educational Robotics to improve inclusion means to enter a network of interacting variables. These variables are both psychological variables, focused on the individual, and psycho-social variables, focused on the individual in context. These variables have effects one on the other and together can determine inclusion that, in turn, can affect school performance or drop-out.

Our paper in this field, proposes to use sociometric measures to assess inclusion. Sociometric tools, widely used in different context including school (Giacomucci and Skolnik, 2021; Casado-Robles et al., 2022), allow to overcome the limit that self-report measures can show in evaluating aspects that depend on other people as social acceptance and inclusion.

On a wider theoretical level, moreover, our results support that constructivism, constructionism, and social constructivism, the theoretical basis ER relies on, can be the framework to face the educational challenges our society is facing.

Nonetheless, this research opens new questions. How long does this positive effect on relations last? Does it have effect on drop-out? What is the weight of individual differences?

These issues will be addressed in future research on Educational Robotics that can be considered an excellent tool to promote positive connections between peers, positive feelings in the people involved, and the acquisition of skills that can be useful in later careers, opening new occupational possibilities.

## Data Availability

The original contributions presented in the study are included in the article/Supplementary Material, further inquiries can be directed to the corresponding author.
