# Supplementary material for: Coding and educational robotics with peers: The C0D1NC experience to foster inclusion
Source: Front Robot AI. 2022 Sep 16;9:825536. doi: 10.3389/frobt.2022.825536 (PMC9523221; doi:10.3389/frobt.2022.825536)
Supplement: Supplementary file 1 [file DataSheet1.pdf]

Appendix

Table of the indexes and sociograms for all groups

<CODINC_

Sociogram Affective and Group pre and post test Youngster

Affective	Nodes	Edges	Average	Density	Coherence	Positive	Negative
Pre test	16,8	132,6	7,768	0,484	0,592	99 (**5,9**)	33,6 (**2**)
Post test	17	154,8	9,118	0,57	0,618	121,6 (**7,15**)	33,2 (**1,95**)
**Group O.**	**Nodes**	**Edges**	**Average**	**Density**	**Coherence**	**Positive**	**Negative**
Pre test	16,8	133,8	8,064	0,514	0,574	98,8 (**5,88**)	35 (**2,08**)
Post test	17	149	9,016	0,576	0,59	121,2 (**7,13**)	27,8 (**1,63**)

Control Group Youngster

Affective	Nodes	Edges	Average	Density	Coherence	Positive	Negative
Pre test	12,7	91,7	7,1	0,6	0,7	67,7 (**5.33**)	24,0 (**1,89**)
Post test	12,7	97,0	7,6	0,6	0,7	73,7 (**5,8**)	23,3 (**1,83**)
**Group O.**	**Nodes**	**Edges**	**Average**	**Density**	**Coherence**	**Positive**	**Negative**
Pre test	12,7	104,3	8,3	0,7	0,7	70,0 (**5,51**)	34,7 (**2,73**)
Post test	12,7	99,7	7,9	0,7	0,7	71,0 (**5,59**)	28,7 (**2,26**)

<CODINC_

Sociogram Affective and Group pre and post test Pupils

Affective	Nodes	Edges	Average	Density	Coherence	Positive	Negative
Pre test	17,2	145,6	8,3	0,5	0,6	76,4 (**4,44**)	69,2 (**4**)
Post test	17,5	157,3	8,8	0,6	0,6	89,1 (**5,09**)	68,2 (**3,89**)
**Group O.**	**Nodes**	**Edges**	**Average**	**Density**	**Coherence**	**Positive**	**Negative**
Pre test	17,2	146,8	8,4	0,5	0,6	81,2 (**4,72**)	65,6 (**3,81**)
Post test	17,2	156,1	9,0	0,6	0,6	87,1 (**5,06**)	69,0 (**4**)

Control Group Pupils

Affective	Nodes	Edges	Average	Density	Coherence	Positive	Negative
Pre test	14,0	133,3	9,1	0,7	0,7	48,7 (**3,48**)	83,0 (**5,93**)
Post test	14,0	120,7	8,3	0,6	0,7	49,0 (**3,5**)	71,7 (**5,12**)
**Group O.**	**Nodes**	**Edges**	**Average**	**Density**	**Coherence**	**Positive**	**Negative**
Pre test	14,0	128,0	8,8	0,7	0,7	53,3 (**3,81**)	74,0 (**5,29**)
Post test	14,0	112,0	7,8	0,6	0,6	49,3 (**3,52**)	62,7 (**4,48**)

Sociogram Affective pre and post test Youngster Group 1

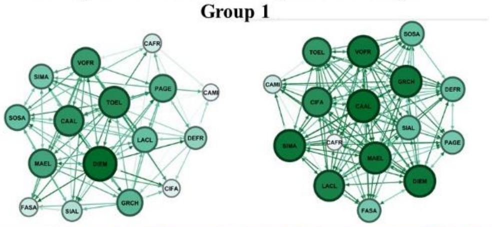

	Nodes	Edges	Average	Density	Coherence	Positive	Negative
Pre test	16	123	7,7	0,51	0,68	73	50
Post test	16	189	11,8	0,79	0,74	162	27
		+ 54%				+ 122%	- 46%

Group Organization pre and post test Youngster Group 1

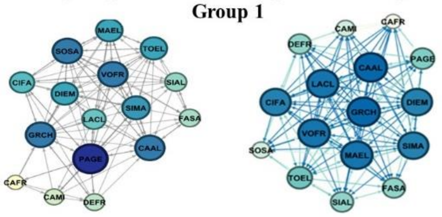

	Nodes	Edges	Average	Density	Coherence	Positive	Negative
Pre test	16	118	7,4	0,49	0,56	74	44
Post test	16	178	11,1	0,74	0,69	165	13
		+ 51%				+ 123%	- 70%

Sociogram Affective pre and post test Youngster Control group

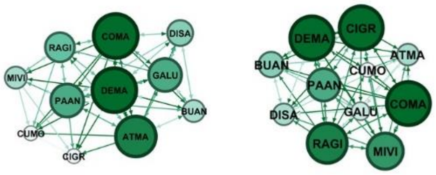

	Nodes	Edges	Average	Density	Coherence	Positive	Negative
Pre test	11	66	6	0,6	0,73	59	7
Post test	11	72	6,5	0,65	0,64	65	7
		+ 9%				+ 10%	=

Sociogram Group Organization pre and post test Youngster Control Group

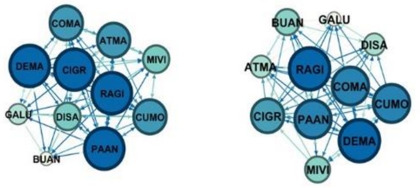

	Nodes	Edges	Average	Density	Coherence	Positive	Negative
Pre test	11	96	8,77	0,87	0,9	72	25
Post test	11	80	7,3	0,7	0,75	63	17
		- 17%				- 12%	- 18%

Sociogram Affective pre / post test - Pupils Group 2

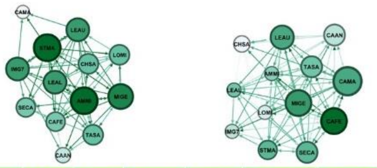

	Nodes	Edges	Average	Density	Coherence	Positive	Negative
Pre test	13	72	5,54	0,46	0,47	32	40
Post test	13	110	8,46	0,71	0,75	62	48
		+ 53%				+ 94%	+ 20%

Sociogram Group Organization pre and post test Group 2

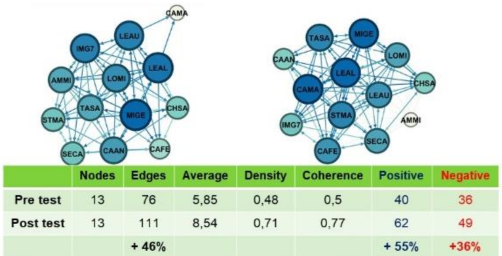

	Nodes	Edges	Average	Density	Coherence	Positive	Negative
Pre test	13	76	5,85	0,48	0,5	40	36
Post test	13	111	8,54	0,71	0,77	62	49
		+ 46%				+ 55%	+36%

Sociogram Affective pre and post test
Group 3

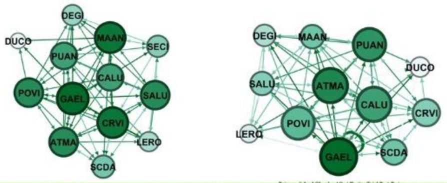

	Nodes	Edges	Average	Density	Coherence	Positive	Negative
Pre test	13	84	6,46	0,54	0,75	46	38
Post test	12	86	7,17	0,65	0,71	58	28
		+ 11%				+ 26%	- 23%

Sociogram Group Organization pre and post test
Group 3

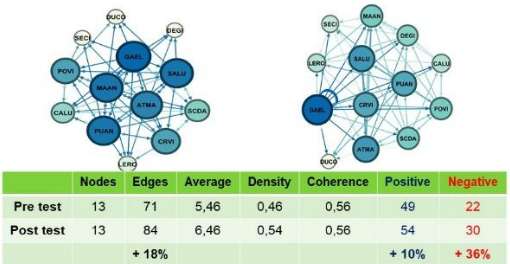

	Nodes	Edges	Average	Density	Coherence	Positive	Negative
Pre test	13	71	5,46	0,46	0,56	49	22
Post test	13	84	6,46	0,54	0,56	54	30
		+ 18%				+ 10%	+ 36%

Sociogram Affective pre and post test Pupils
Group 4

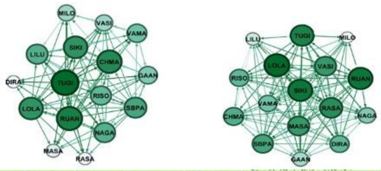

	Nodes	Edges	Average	Density	Coherence	Positive	Negative
Pre test	16	109	6,81	0,54	0,47	52	57
Post test	16	192	12	0,8	0,84	125	67
		+ 76%				+ 140%	+ 18%

Sociogram Group Organization pre and post test Pupils
Group 4

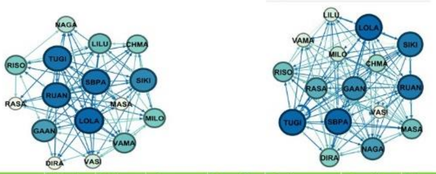

	Nodes	Edges	Average	Density	Coherence	Positive	Negative
Pre test	16	123	7,69	0,51	0,63	81	42
Post test	16	195	12,2	0,81	0,79	124	71
		+ 59%				+ 53%	+ 65%

Sociogram Affective pre and post test Pupils
Group 5

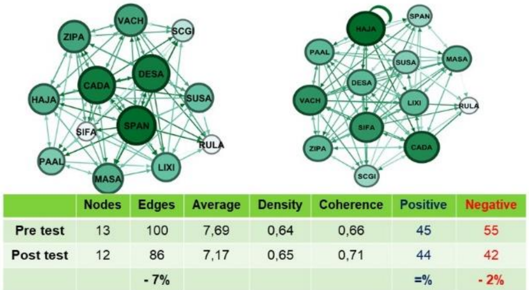

	Nodes	Edges	Average	Density	Coherence	Positive	Negative
Pre test	13	100	7,69	0,64	0,66	45	55
Post test	12	86	7,17	0,65	0,71	44	42
		- 7%				=%	- 2%

Sociogram Group Organization pre and post test Pupils
Group 5

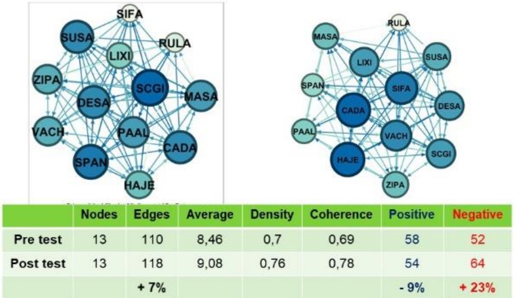

	Nodes	Edges	Average	Density	Coherence	Positive	Negative
Pre test	13	110	8,46	0,7	0,69	58	52
Post test	13	118	9,08	0,76	0,78	54	64
		+ 7%				- 9%	+ 23%

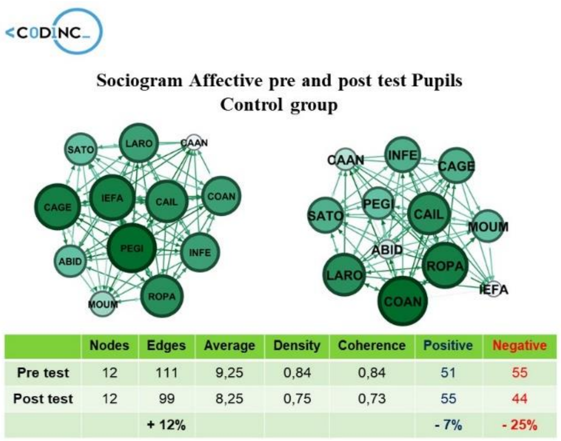

	Nodes	Edges	Average	Density	Coherence	Positive	Negative
Pre test	12	111	9,25	0,84	0,84	51	55
Post test	12	99	8,25	0,75	0,73	55	44
		+ 12%				- 7%	- 25%

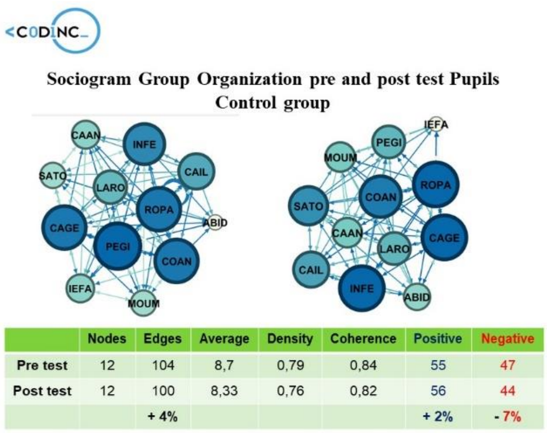

	Nodes	Edges	Average	Density	Coherence	Positive	Negative
Pre test	12	104	8,7	0,79	0,84	55	47
Post test	12	100	8,33	0,76	0,82	56	44
		+ 4%				+ 2%	- 7%
